# Effectiveness of 2-Dose Vaccination with mRNA COVID-19 Vaccines Against COVID-19–Associated Hospitalizations Among Immunocompromised Adults — Nine States, January–September 2021

**DOI:** 10.15585/mmwr.mm7044e3

**Published:** 2021-11-05

**Authors:** Peter J. Embi, Matthew E. Levy, Allison L. Naleway, Palak Patel, Manjusha Gaglani, Karthik Natarajan, Kristin Dascomb, Toan C. Ong, Nicola P. Klein, I-Chia Liao, Shaun J. Grannis, Jungmi Han, Edward Stenehjem, Margaret M. Dunne, Ned Lewis, Stephanie A. Irving, Suchitra Rao, Charlene McEvoy, Catherine H. Bozio, Kempapura Murthy, Brian E. Dixon, Nancy Grisel, Duck-Hye Yang, Kristin Goddard, Anupam B. Kharbanda, Sue Reynolds, Chandni Raiyani, William F. Fadel, Julie Arndorfer, Elizabeth A. Rowley, Bruce Fireman, Jill Ferdinands, Nimish R. Valvi, Sarah W. Ball, Ousseny Zerbo, Eric P. Griggs, Patrick K. Mitchell, Rachael M. Porter, Salome A. Kiduko, Lenee Blanton, Yan Zhuang, Andrea Steffens, Sarah E. Reese, Natalie Olson, Jeremiah Williams, Monica Dickerson, Meredith McMorrow, Stephanie J. Schrag, Jennifer R. Verani, Alicia M. Fry, Eduardo Azziz-Baumgartner, Michelle A. Barron, Mark G. Thompson, Malini B. DeSilva

**Affiliations:** ^1^Regenstrief Institute, Indianapolis, Indiana; ^2^Indiana University School of Medicine, Indianapolis, Indiana; ^3^Westat, Rockville, Maryland; ^4^Center for Health Research, Kaiser Permanente Northwest, Portland, Oregon; ^5^CDC COVID-19 Response Team; ^6^Baylor Scott & White Health, Texas A&M University College of Medicine, Temple, Texas; ^7^Department of Biomedical Informatics, Columbia University, New York, New York; ^8^New York Presbyterian Hospital, New York, New York; ^9^Division of Infectious Diseases and Clinical Epidemiology, Intermountain Healthcare, Salt Lake City, Utah; ^10^School of Medicine, University of Colorado, Anschutz Medical Campus, Aurora, Colorado; ^11^Kaiser Permanente Vaccine Study Center, Kaiser Permanente Northern California, Oakland, California; ^12^Center for Biomedical Informatics, Regenstrief Institute, Indianapolis, Indiana; ^13^HealthPartners Institute, Minneapolis, Minnesota; ^14^Fairbanks School of Public Health, Indiana University, Indianapolis, Indiana; ^15^Children’s Minnesota, Minneapolis, Minnesota.

Immunocompromised persons, defined as those with suppressed humoral or cellular immunity resulting from health conditions or medications, account for approximately 3% of the U.S. adult population (*1*). Immunocompromised adults are at increased risk for severe COVID-19 outcomes (*2*) and might not acquire the same level of protection from COVID-19 mRNA vaccines as do immunocompetent adults (*3*,*4*). To evaluate vaccine effectiveness (VE) among immunocompromised adults, data from the VISION Network* on hospitalizations among persons aged ≥18 years with COVID-19–like illness from 187 hospitals in nine states during January 17–September 5, 2021 were analyzed. Using selected discharge diagnoses,^†^ VE against COVID-19–associated hospitalization conferred by completing a 2-dose series of an mRNA COVID-19 vaccine ≥14 days before the index hospitalization date^§^ (i.e., being fully vaccinated) was evaluated using a test-negative design comparing 20,101 immunocompromised adults (10,564 [53%] of whom were fully vaccinated) and 69,116 immunocompetent adults (29,456 [43%] of whom were fully vaccinated). VE of 2 doses of mRNA COVID-19 vaccine against COVID-19–associated hospitalization was lower among immunocompromised patients (77%; 95% confidence interval [CI] = 74%–80%) than among immunocompetent patients (90%; 95% CI = 89%–91%). This difference persisted irrespective of mRNA vaccine product, age group, and timing of hospitalization relative to SARS-CoV-2 (the virus that causes COVID-19) B.1.617.2 (Delta) variant predominance in the state of hospitalization. VE varied across immunocompromising condition subgroups, ranging from 59% (organ or stem cell transplant recipients) to 81% (persons with a rheumatologic or inflammatory disorder). Immunocompromised persons benefit from mRNA COVID-19 vaccination but are less protected from severe COVID-19 outcomes than are immunocompetent persons, and VE varies among immunocompromised subgroups. Immunocompromised persons receiving mRNA COVID-19 vaccines should receive 3 doses and a booster, consistent with CDC recommendations (*5*), practice nonpharmaceutical interventions, and, if infected, be monitored closely and considered early for proven therapies that can prevent severe outcomes.

Data came from the VISION Network, a collaboration between CDC and seven U.S. health care systems and research centers with integrated medical, laboratory, and vaccination records that was established to assess the effectiveness of COVID-19 vaccines (*6*). Eligible hospitalizations were defined as those among adults aged ≥18 years with SARS-CoV-2 molecular testing (from 14 days before through 72 hours after admission) and a COVID-19–like illness discharge diagnosis.^¶^ Encounters without molecular testing were excluded from this analysis. Immunocompromised patients were defined by the presence of at least one selected discharge diagnosis for immunocompromising conditions using the *International Classification of Diseases, Ninth Revision *and *International Classification of Diseases, Tenth Revision*. Diagnoses across five categories of immunocompromising conditions were derived from lists used in previous studies of large hospital-based or administrative databases and included the following conditions: 1) solid malignancies, 2) hematologic malignancies, 3) rheumatologic or inflammatory disorders, 4) other intrinsic immune conditions or immunodeficiencies, and 5) organ or stem cell transplants (*7**–**9*). Immunosuppressive medication use data were not available for these analyses. Vaccination status was documented in electronic health records or state immunization registries (*6*). Full vaccination was defined as receipt of the second in a 2-dose series of Moderna (mRNA-1273) or Pfizer-BioNTech (BNT162b2) vaccines ≥14 days before the index hospitalization date; unvaccinated patients had not received any COVID-19 vaccine doses and were considered the referent group. Patients who received 1) only 1 mRNA vaccine dose, 2) ≥3 mRNA vaccine doses, 3) the second dose <14 days before index hospitalization date, or 4) the Janssen (Johnson & Johnson [Ad26.COV2]) vaccine were excluded from the analysis.

VE was estimated using a test-negative design comparing the odds of a positive test result for SARS-CoV-2 between fully vaccinated and unvaccinated patients using multivariable logistic regression models. VE was adjusted for age, geographic region, calendar time (days from January 1 to hospitalization), and local virus circulation in the community where each partner site was located and weighted for inverse propensity to be vaccinated or unvaccinated (calculated separately for each VE estimate). Generalized boosted regression trees were used to estimate the propensity to be vaccinated based on sociodemographic characteristics, underlying medical conditions, known previous SARS-CoV-2 infection, and hospital characteristics. VE estimates were stratified by immunocompromised status, mRNA COVID-19 vaccine product received, age group, and network partner (representing different health care systems and geographic regions). Among immunocompromised patients, VE was also calculated separately among subgroups of patients with each of the five categories of immunocompromising diagnoses (subgroups that were not mutually exclusive). VE was also calculated separately for hospitalizations occurring before and after the period when the Delta variant accounted for ≥50% of sequenced isolates within each site’s state (*10*). VE estimates with 95% CIs that did not overlap were considered statistically different, which is a conservative approach. This study was reviewed and approved by Westat, Inc. institutional review board.**

Among 69,116 immunocompetent adults and 20,101 immunocompromised adults hospitalized with COVID-19–like illness and with available molecular testing results for SARS-CoV-2, 29,456 (43%) and 10,564 (53%), respectively, were fully vaccinated (Table 1). The median ages of immunocompetent and immunocompromised patients were 68 years (interquartile range [IQR] = 52–79 years) and 70 years (IQR = 60–78 years), respectively. Among immunocompetent patients, 42% had received the Moderna vaccine and 58% had received the Pfizer-BioNTech vaccine, and among immunocompromised patients, 41% and 59% had received Moderna and Pfizer-BioNTech vaccines, respectively. Among immunocompetent patients, the median interval from receipt of the second vaccine dose to hospital admission was 89 days (IQR = 52–129 days) among Moderna vaccine recipients and 90 days (IQR = 52–131 days) among Pfizer-BioNTech vaccine recipients; among immunocompromised patients, the intervals were 89 days (IQR = 52–128 days) and 89 days (IQR = 53–128 days) for Moderna vaccine and Pfizer-BioNTech vaccine recipients, respectively.

**TABLE 1:** Characteristics of COVID-19–like illness hospitalizations* among immunocompetent and immunocompromised adults aged ≥18 years and proportions of 2-dose mRNA COVID-19 vaccine recipients with laboratory-confirmed SARS-CoV-2 infection — nine states,^†^ January–September 2021

Characteristic	Immunocompetent					Immunocompromised^§^				
	Total	Vaccinated^¶^		SARS-CoV-2–positive test result		Total	Vaccinated^¶^		SARS-CoV-2–positive test result	
	No. (column %)	No. (row %)	SMD**	No. (row %)	SMD^††^	No. (column %)	No. (row %)	SMD**	No. (row %)	SMD^††^
**All hospitalizations**	**69,116 (100)**	**29,456 (43)**	**—**	**10,961 (16)**	**—**	**20,101 (100)**	**10,564 (53)**	**—**	**1,537 (8)**	**—**
**Site**										
Columbia University	4,221 (6)	1,338 (32)	0.70	673 (16)	0.39	1,615 (8)	645 (40)	0.86	149 (9)	0.50
HealthPartners	1,695 (2)	1,022 (60)		100 (6)		721 (4)	467 (65)		24 (3)	
Intermountain Healthcare	6,937 (10)	2,501 (36)		1,925 (28)		1,479 (7)	659 (45)		292 (20)	
Kaiser Permanente Northern California	22,331 (32)	14,222 (64)		3,253 (15)		7,518 (37)	5,707 (76)		461 (6)	
Kaiser Permanente Northwest	3,531 (5)	1,716 (49)		347 (10)		1,117 (6)	614 (55)		47 (4)	
Regenstrief Institute	19,099 (28)	6,188 (32)		3,562 (19)		3,000 (15)	1,121 (37)		320 (11)	
University of Colorado	11,302 (16)	2,469 (22)		1,101 (10)		4,651 (23)	1,351 (29)		244 (5)	
**Age group, yrs**										
18–49	15,891 (23)	3,469 (22)	0.62	3,542 (22)	0.43	2,291 (11)	709 (31)	0.47	241 (11)	0.21
50–64	13,669 (20)	4,597 (34)		2,989 (22)		4,524 (23)	1,874 (41)		411 (9)	
65–74	15,715 (23)	7,477 (48)		2,101 (13)		6,149 (31)	3,429 (56)		431 (7)	
75–84	14,421 (21)	8,270 (57)		1,491 (10)		5,064 (25)	3,201 (63)		341 (7)	
≥85	9,420 (14)	5,643 (60)		838 (9)		2,073 (10)	1,351 (65)		113 (5)	
**Sex**										
Men^§§^	30,625 (44)	13,106 (43)	–0.01	5,406 (18)	–0.12	9,552 (48)	5,082 (53)	–0.02	762 (8)	–0.04
Women	38,491 (56)	16,350 (42)		5,555 (14)		10,549 (52)	5,482 (52)		775 (7)	
**Race, (regardless of ethnicity)**										
White	45,206 (65)	20,094 (44)	0.28	6,512 (14)	0.20	13,834 (69)	7,344 (53)	0.23	985 (7)	0.16
Black	7,204 (10)	2,107 (29)		1,274 (18)		1,821 (9)	819 (45)		182 (10)	
Other	5,382 (8)	3,126 (58)		722 (13)		1,725 (9)	1,164 (67)		110 (6)	
Unknown	11,324 (16)	4,129 (36)		2,453 (22)		2,721 (14)	1,237 (45)		260 (10)	
**Ethnicity**										
Hispanic	9,415 (14)	3,464 (37)	0.10	2,069 (22)	0.26	2,786 (14)	1,366 (49)	0.09	271 (10)	0.14
Non-Hispanic	48,146 (70)	20,753 (43)		6,498 (13)		14,448 (72)	7,544 (52)		1,020 (7)	
Unknown	11,555 (17)	5,239 (45)		2,394 (21)		2,867 (14)	1,654 (58)		246 (9)	
**Chronic respiratory condition** ^¶¶^										
Has chronic respiratory condition	44,264 (64)	19,788 (45)	0.11	6,891 (16)	–0.03	13,652 (68)	7,331 (54)	0.07	1,084 (8)	0.06
No chronic respiratory condition^§§^	24,852 (36)	9,668 (39)		4,070 (16)		6,449 (32)	3,233 (50)		453 (7)	
**ICU admission**										
Admitted to ICU	10,939 (16)	4,278 (39)	–0.06	1,700 (16)	–0.01	4,285 (21)	1,977 (46)	–0.13	361 (8)	0.06
Not admitted to ICU^§§^	58,177 (84)	25,178 (43)		9,261 (16)		15,816 (79)	8,587 (54)		1,176 (7)	
**COVID-19 vaccination status**										
Unvaccinated	39,660 (57)	0 (—)	—	9,853 (25)	0.94	9,537 (47)	0 (—)	—	1,127 (12)	0.60
Moderna (mRNA-1273)	12,341 (18)	12,341 (100)		357 (3)		4,337 (22)	4,337 (100)		138 (3)	
Pfizer-BioNTech (BNT162b2)	17,115 (25)	17,115 (100)		751 (4)		6,227 (31)	6,227 (100)		272 (4)	

**Abbreviations:** ICD-9 = *International Classification of Diseases, Ninth Revision*; ICD-10 = *International Classification of Diseases, Tenth Revision*; ICU = intensive care unit; SMD = standardized mean or proportion difference.

* Hospitalizations with a discharge code consistent with COVID-19–like illness were included, such as acute respiratory illness (e.g., COVID-19, respiratory failure, or pneumonia) or related signs or symptoms (cough, fever, dyspnea, vomiting, or diarrhea), using diagnosis codes from ICD-9 and ICD-10. Clinician-ordered molecular assays (e.g., real-time reverse transcription–polymerase chain reaction test) for SARS-CoV-2 occurring ≤14 days before to <72 hours after hospital admission were included.

^†^ Partners contributing data on hospitalizations were in California (range of earliest to latest hospitalization: March 1–September 5), Colorado (January 22–August 31), Indiana (January 22–September 5), Minnesota and Wisconsin (January 17–August 18), New York (January 22–September 5), Oregon and Washington (February 1–August 20), and Utah (February 1–September 5).

^§^ Immunocompromised status was presumed based on the presence of at least one discharge diagnosis, using ICD-9 and ICD-10 diagnosis codes for solid malignancy (ICD-10 codes: C00–C80, C7A, C7B, D3A, Z51.0, and Z51.1), hematologic malignancy (ICD-10 codes: C81–C86, C88, C90–C96, D46, D61.0, D70.0, D61.2, D61.9, and D71), rheumatologic or inflammatory disorder (ICD-10 codes: D86, E85 [except E85.0], G35, J67.9, L40.54, L40.59, L93.0, L93.2, L94, M05–M08, M30, M31.3, M31.5, M32–M34, M35.3, M35.8, M35.9, M46, and T78.40), other intrinsic immune condition or immunodeficiency (ICD-10 codes: D27.9, D61.09, D72.89, D80, D81 [except D81.3], D82–D84, D89 [except D89.2], K70.3, K70.4, K72, K74.3–K74.6 [except K74.60 and K74.69], N04, and R18), or organ or stem cell transplant (ICD-10 codes: T86 [except T86.82–T86.84, T86.89, and T86.9], D47.Z1, Z48.2, Z94, and Z98.85).

^¶^ Vaccination was defined as having received exactly 2 doses of an mRNA-based COVID-19 vaccine ≥14 days before the hospitalization index date, which was the date of respiratory specimen collection associated with the most recent positive or negative SARS-CoV-2 test result before the hospitalization or the hospitalization date if testing only occurred after the admission.

** An absolute standardized mean or proportion difference ≥0.10 indicates a nonnegligible difference in variable distributions between hospitalizations for vaccinated versus unvaccinated patients.

^††^ An absolute standardized mean or proportion difference ≥0.10 indicates a nonnegligible difference in variable distributions between hospitalizations for SARS-CoV-2–positive versus SARS-CoV-2–negative patients.

^§§^ Indicates the reference group used for standardized mean or proportion difference calculations for dichotomous variables.

^¶¶^ Chronic respiratory condition was defined as the presence of discharge code for asthma, chronic obstructive pulmonary disease, or other lung disease using diagnosis codes from ICD-9 and ICD-10.

Among immunocompetent patients, SARS-CoV-2 infection was laboratory-confirmed in 9,853 (24.8%) unvaccinated and 1,108 (3.8%) fully vaccinated persons, compared with 1,127 (11.8%) unvaccinated and 410 (3.9%) fully vaccinated immunocompromised patients (Table 2). VE of 2 doses of mRNA vaccine against COVID-19 hospitalization was lower among immunocompromised patients (77%) than among immunocompetent patients (90%). Differences persisted when analyzed among patients aged 18–64 years and aged ≥65 years, and among Moderna and Pfizer-BioNTech vaccine recipients. Among immunocompromised patients, VE was 81% for the Moderna vaccine and 71% for the Pfizer-BioNTech vaccine; however, CIs slightly overlapped between these two estimates. VE was similarly lower among immunocompromised than among immunocompetent patients both before the period of Delta variant predominance (76%; 95% CI = 69%–81% versus 91%; 95% CI = 90%–93%) and during the period of Delta variant predominance (79%; 95% CI = 74%–83% versus 90%; 95% CI = 89%–91%). Across network partners, VE point estimates varied more for immunocompromised patients (57–85%) than for immunocompetent patients (84%–94%).

**TABLE 2:** Two-dose mRNA COVID-19 vaccine effectiveness* against laboratory-confirmed COVID-19–associated hospitalization^†^ among immunocompetent and immunocompromised adults aged ≥18 years, by age group and vaccine — nine states,^§^ January–September 2021

Age group, yrs, vaccine	Total no. of adults	SARS-CoV-2–positive test result, no. (row %)	VE,^¶^ % (95% CI)
**≥18, any mRNA COVID-19 vaccine**			
**Immunocompetent (n = 69,116)**			
Unvaccinated	**39,660**	9,853 (24.8)	Ref
Vaccinated with 2 doses**	**29,456**	1,108 (3.8)	90 (89–91)
**Immunocompromised**^††^ **(n = 20,101)**			
Unvaccinated	**9,537**	1,127 (11.8)	Ref
Vaccinated with 2 doses**	**10,564**	410 (3.9)	77 (74–80)
**18–64, any mRNA COVID-19 vaccine**			
**Immunocompetent (n = 29,560)**			
Unvaccinated	**21,494**	6,243 (29.1)	Ref
Vaccinated with 2 doses**	**8,066**	288 (3.6)	93 (92–94)
**Immunocompromised**^††^ **(n = 6,815)**			
Unvaccinated	**4,232**	544 (12.8)	Ref
Vaccinated with 2 doses**	**2,583**	108 (4.2)	80 (74–84)
**≥65, any mRNA COVID-19 vaccine**			
**Immunocompetent (n = 39,556)**			
Unvaccinated	**18,166**	3,610 (19.9)	Ref
Vaccinated with 2 doses**	**21,390**	820 (3.8)	87 (86–88)
**Immunocompromised**^††^ **(n = 13,286)**			
Unvaccinated	**5,305**	583 (11)	Ref
Vaccinated with 2 doses**	**7,981**	302 (3.8)	75 (70–79)
**≥18, Moderna (mRNA-1273) vaccine**			
**Immunocompetent (n = 52,001)**			
Unvaccinated	**39,660**	9,853 (24.8)	Ref
Vaccinated with 2 doses**	**12,341**	357 (2.9)	93 (92–94)
**Immunocompromised**^††^ **(n = 13,874)**			
Unvaccinated	**9,537**	1,127 (11.8)	Ref
Vaccinated with 2 doses**	**4,337**	138 (3.2)	81 (76–85)
**≥18, Pfizer-BioNTech (BNT162b2) vaccine**			
**Immunocompetent (n = 56,775)**			
Unvaccinated	**39,660**	9,853 (24.8)	Ref
Vaccinated with 2 doses**	**17,115**	751 (4.4)	88 (86–89)
**Immunocompromised**^††^ **(n = 15,764)**			
Unvaccinated	**9,537**	1,127 (11.8)	Ref
Vaccinated with 2 doses**	**6,227**	272 (4.4)	71 (65–76)

**Abbreviations:** CI = confidence interval; ICD-9 = *International Classification of Diseases, Ninth Revision*; ICD-10 = *International Classification of Diseases, Tenth Revision*; Ref = referent group; VE = vaccine effectiveness.

* VE was estimated using a test-negative design, adjusted for age, geographic region, calendar time (days since January 1, 2021), and local virus circulation (percentage of SARS-CoV-2–positive results from testing within the counties surrounding the facility on the date of the hospitalization) and weighted for inverse propensity to be vaccinated or unvaccinated (calculated separately for each VE estimate) using sociodemographic characteristics, underlying medical conditions, known previous SARS-CoV-2 infection, and hospital characteristics, in addition to age, geographic region, calendar time, and local virus circulation.

^†^ Hospitalizations with a discharge code consistent with COVID-19–like illness were included, such as acute respiratory illness (e.g., COVID-19, respiratory failure, or pneumonia) or related signs or symptoms (cough, fever, dyspnea, vomiting, or diarrhea), using diagnosis codes from ICD-9 and ICD-10. Clinician-ordered molecular assays (e.g., real-time reverse transcription–polymerase chain reaction test) for SARS-CoV-2 occurring ≤14 days before to <72 hours after hospital admission were included.

^§^ Partners contributing data on hospitalizations were in California (range of earliest to latest hospitalization: March 1–September 5), Colorado (January 22–August 31), Indiana (January 22–September 5), Minnesota and Wisconsin (January 17–August 18), New York (January 22–September 5), Oregon and Washington (February 1–August 20), and Utah (February 1–September 5).

^¶^ VE was calculated as [1−odds ratio]x100%.

** Vaccination was defined as having received exactly 2 doses of an mRNA-based COVID-19 vaccine ≥14 days before the hospitalization index date, which was the date of respiratory specimen collection associated with the most recent positive or negative SARS-CoV-2 test result before the hospitalization or the hospitalization date if testing only occurred after the admission.

^††^ Immunocompromised status was presumed based on the presence of at least one discharge diagnosis, using ICD-9 and ICD-10 diagnosis codes for solid malignancy (ICD-10 codes: C00–C80, C7A, C7B, D3A, Z51.0, and Z51.1), hematologic malignancy (ICD-10 codes: C81–C86, C88, C90–C96, D46, D61.0, D70.0, D61.2, D61.9, and D71), rheumatologic or inflammatory disorder (ICD-10 codes: D86, E85 [except E85.0], G35, J67.9, L40.54, L40.59, L93.0, L93.2, L94, M05–M08, M30, M31.3, M31.5, M32–M34, M35.3, M35.8, M35.9, M46, and T78.40), other intrinsic immune condition or immunodeficiency (ICD-10 codes: D27.9, D61.09, D72.89, D80, D81 [except D81.3], D82–D84, D89 [except D89.2], K70.3, K70.4, K72, K74.3–K74.6 [except K74.60 and K74.69], N04, and R18), or organ or stem cell transplant (ICD-10 codes: T86 [except T86.82–T86.84, T86.89, and T86.9], D47.Z1, Z48.2, Z94, and Z98.85).

Among immunocompromised patients, 8,887 (44%) had a solid malignancy, (range across network partners = 34%–47%), 2,790 (14%) a hematologic malignancy (range = 11%–19%), 5,024 (25%) a rheumatologic or inflammatory disorder (range = 22%–30%), 6,380 (32%) another intrinsic immune condition or immunodeficiency (range = 29%–37%), and 1,416 (7%) had received an organ or stem cell transplant (range = 4%–10%). VE point estimates ranged from 59% (organ or stem cell transplant patients) to 81% (patients with a rheumatologic or inflammatory disorder) across these subgroups (Table 3). Subgroup VE point estimates were generally higher for the Moderna vaccine than for the Pfizer-BioNTech vaccine but were the same among patients with a rheumatologic or inflammatory disorder.

**TABLE 3:** Two-dose mRNA COVID-19 vaccine effectiveness* against laboratory-confirmed COVID-19–associated hospitalization^†^ among subgroups of adults aged ≥18 years with specific types of conditions and presumed to be immunocompromised (20,101)^§^ — nine states,^¶^ January–September 2021

Condition (no. of adults)	Total	SARS-CoV-2–positive tests, no. (row %)	VE,** % (95% CI)
**Solid malignancy**^††^ **(8,887)**			
Unvaccinated	**3,986**	304 (7.6)	Ref
Vaccinated with any 2 mRNA vaccine doses^§§^	**4,901**	106 (2.2)	79 (73–84)
Vaccinated with 2 Moderna (mRNA-1273) vaccine doses^§§^	**2,053**	30 (1.5)	85 (76–91)
Vaccinated with 2 Pfizer-BioNTech (BNT162b2) vaccine doses^§§^	**2,848**	76 (2.7)	72 (62–80)
**Hematologic malignancy**^¶¶^ **(2,790)**			
Unvaccinated	**1,156**	130 (11.2)	Ref
Vaccinated with any 2 mRNA vaccine doses^§§^	**1,634**	86 (5.3)	74 (62–83)
Vaccinated with 2 Moderna vaccine doses^§§^	**660**	26 (3.9)	85 (74–92)
Vaccinated with 2 Pfizer-BioNTech vaccine doses^§§^	**974**	60 (6.2)	62 (42–75)
Rheumatologic or inflammatory disorder*** (5,024)			
Unvaccinated	**2,380**	383 (16.1)	Ref
Vaccinated with any 2 mRNA vaccine doses^§§^	**2,644**	123 (4.6)	81 (75–86)
Vaccinated with 2 Moderna vaccine doses^§§^	**1,053**	48 (4.6)	78 (65–86)
Vaccinated with 2 Pfizer-BioNTech vaccine doses^§§^	**1,591**	75 (4.7)	78 (69–84)
**Other intrinsic immune condition or immunodeficiency^†††^ (6,380)**			
Unvaccinated	**3,418**	429 (12.6)	Ref
Vaccinated with any 2 mRNA vaccine doses^§§^	**2,962**	137 (4.6)	73 (66–80)
Vaccinated with 2 Moderna vaccine doses^§§^	**1,199**	42 (3.5)	81 (71–87)
Vaccinated with 2 Pfizer-BioNTech vaccine doses^§§^	**1,763**	95 (5.4)	64 (50–74)
**Organ or stem cell transplant**^§§§^ **(1,416)**			
Unvaccinated	**607**	92 (15.2)	Ref
Vaccinated with any 2 mRNA vaccine doses^§§^	**809**	80 (9.9)	59 (38–73)
Vaccinated with 2 Moderna vaccine doses^§§^	**337**	31 (9.2)	70 (46–83)
Vaccinated with 2 Pfizer-BioNTech vaccine doses^§§^	**472**	49 (10.4)	45 (13–66)

**Abbreviations:** CI = confidence interval; ICD-9 = *International Classification of Diseases, Ninth Revision*; ICD-10 = *International Classification of Diseases, Tenth Revision*; Ref = referent group; VE = vaccine effectiveness.

* VE was estimated using a test-negative design, adjusted for age, geographic region, calendar time (days since January 1, 2021), and local virus circulation (percentage of SARS-CoV-2–positive results from testing within the counties surrounding the facility on the date of the hospitalization) and weighted for inverse propensity to be vaccinated or unvaccinated (calculated separately for each VE estimate) using sociodemographic characteristics, underlying medical conditions, known previous SARS-CoV-2 infection, and hospital characteristics, in addition to age, geographic region, calendar time, and local virus circulation.

^†^ Hospitalizations with a discharge code consistent with COVID-19–like illness were included, such as acute respiratory illness (e.g., COVID-19, respiratory failure, or pneumonia) or related signs or symptoms (cough, fever, dyspnea, vomiting, or diarrhea), using diagnosis codes from ICD-9 and ICD-10. Clinician-ordered molecular assays (e.g., real-time reverse transcription–polymerase chain reaction test) for SARS-CoV-2 occurring ≤14 days before to <72 hours after hospital admission were included.

^§^ Immunocompromising condition subgroups were not mutually exclusive, and patients could be represented in more than one of the five subgroups (i.e., solid malignancy, hematologic malignancy, rheumatologic or inflammatory disorder, other intrinsic immune condition or immunodeficiency, and organ or stem cell transplant).

^¶^ Partners contributing data on hospitalizations were in California (range of earliest to latest hospitalization: March 1–September 5), Colorado (January 22–August 31), Indiana (January 22–September 5), Minnesota and Wisconsin (January 17–August 18), New York (January 22–September 5), Oregon and Washington (February 1–August 20), and Utah (February 1–September 5).

** VE was calculated as [1−odds ratio]x100%.

^††^ Solid malignancy was defined as the presence of at least one discharge diagnosis using ICD-9 and ICD-10 diagnosis codes. ICD-10 codes included C00–C80, C7A, C7B, D3A, Z51.0, and Z51.1.

^§§^ Vaccination was defined as having received exactly 2 doses of an mRNA-based COVID-19 vaccine ≥14 days before the hospitalization index date, which was the date of respiratory specimen collection associated with the most recent positive or negative SARS-CoV-2 test result before the hospitalization or the hospitalization date if testing only occurred after the admission.

^¶¶^ Hematologic malignancy was defined as the presence of at least one discharge diagnosis using ICD-9 and ICD-10 diagnosis codes. ICD-10 codes included C81–C86, C88, C90–C96, D46, D61.0, D70.0, D61.2, D61.9, and D71.

*** Rheumatologic or inflammatory disorder was defined as the presence of at least one discharge diagnosis using ICD-9 and ICD-10 diagnosis codes. ICD-10 codes included D86, E85 (except E85.0), G35, J67.9, L40.54, L40.59, L93.0, L93.2, L94, M05–M08, M30, M31.3, M31.5, M32–M34, M35.3, M35.8, M35.9, M46, and T78.40.

^†††^ Other intrinsic immune condition or immunodeficiency was defined as the presence of at least one discharge diagnosis using ICD-9 and ICD-10 diagnosis codes. ICD-10 codes included D27.9, D61.09, D72.89, D80, D81 (except D81.3), D82–D84, D89 (except D89.2), K70.3, K70.4, K72, K74.3–K74.6 (except K74.60 and K74.69), N04, and R18.

^§§§^ Organ or stem cell transplant was defined as the presence of at least one discharge diagnosis using ICD-9 and ICD-10 diagnosis codes. ICD-10 codes included T86 (except T86.82–T86.84, T86.89, and T86.9), D47.Z1, Z48.2, Z94, and Z98.85.

## Discussion

In a multistate analysis of approximately 89,000 hospitalizations of adults with COVID-19–like illness during January 17–September 5, 2021, receipt of 2 doses of mRNA COVID-19 vaccine was effective in preventing laboratory-confirmed COVID-19 hospitalizations among patients who were immunocompromised (VE = 77%) and those who were immunocompetent (VE = 90%). Nonetheless, immunocompromised patients were significantly less protected from severe COVID-19 outcomes compared with immunocompetent patients, supporting the recommendation for administration of a third dose of mRNA vaccine to further enhance protection of moderately to severely immunocompromised persons against severe COVID-19 outcomes (*5*).

This study also found that VE was lower among certain subgroups of immunocompromised adults, such as solid organ or stem cell transplant recipients, than among others. These findings are consistent with other studies suggesting that certain immunocompromised persons experience an attenuated immune response to COVID-19 vaccines and make up a large proportion of hospitalizations for infections after vaccination (*3*,*4*). The prevalence of SARS-CoV-2 infection was approximately two times greater among unvaccinated immunocompetent patients compared with unvaccinated immunocompromised patients. Because the study sample was restricted to patients hospitalized with COVID-19–like illness, this difference might be related to a variation in the prevalence of other respiratory virus infections between the two groups, although this is unable to be confirmed. A strength of the test-negative design is that such a difference is not expected to influence the validity of VE estimates stratified by immunocompromised status. 

The findings in this report are subject to at least six limitations. First, the use of selected discharge diagnoses as surrogates for presumed immunocompromised status and the absence of medication and other relevant data might have led to classification of persons as immunocompromised who were not; the opposite is also possible but is less likely. Second, selection bias might be possible if vaccination status influences the likelihood of receiving testing although a previous VISION Network study indicated that vaccination status did not affect receipt of testing (*6*). Third, despite the high specificity of COVID-19 vaccination status from these data sources, misclassification might be possible. Fourth, although inverse weights balanced unvaccinated and vaccinated hospitalized patients on sociodemographic and health characteristics, and further adjustments for age, geographic region, calendar time, and local virus circulation were made, unmeasured and residual confounding (e.g., mask-wearing and waning immunity) in this observational study might have biased these estimates. Fifth, the study only assessed mRNA COVID-19 vaccines and not the Janssen vaccine and included health care systems in only nine states, limiting the potential for the findings of this study to be extrapolated. Finally, immunocompromising conditions were not mutually exclusive, and sparse data in smaller immunocompromised subgroups reduced VE precision, so it was not possible to determine the independent effect of each subgroup on VE.

Immunocompromised persons benefit from and should receive COVID-19 vaccines. Given that VE is lower compared to immunocompetent patients, immunocompromised persons receiving mRNA vaccines should receive 3 doses and a booster 6 months after the third dose, consistent with CDC recommendations (*5*). In addition to vaccination, immunocompromised persons should implement nonpharmaceutical prevention strategies such as masking to help prevent SARS-CoV-2 infection, and, if infected with SARS-CoV-2, be monitored closely and considered early for proven therapies that might prevent progression to severe illness (e.g., monoclonal antibodies). Additional studies are needed to further characterize variation in VE among immunocompromised subpopulations and across geographic regions, determine the degree of improvements in VE conferred by additional COVID-19 vaccine doses in immunocompromised populations, evaluate whether different approaches to vaccine administration might improve VE (e.g., dosage timing or temporarily withholding immunosuppressants), and further evaluate possible differences in VE between vaccine products.

**Summary**

**What is already known about this topic?**

Studies suggest that immunocompromised persons who receive COVID-19 vaccination might not develop high neutralizing antibody titers or be as protected against severe COVID-19 outcomes as are immunocompetent persons.

**What is added by this report?**

Effectiveness of mRNA vaccination against laboratory-confirmed COVID-19–associated hospitalization was lower (77%) among immunocompromised adults than among immunocompetent adults (90%). Vaccine effectiveness varied considerably among immunocompromised patient subgroups.

**What are the implications for public health practice?**

Immunocompromised persons benefit from COVID-19 mRNA vaccination but are less protected from severe COVID-19 outcomes than are immunocompetent persons. Immunocompromised persons receiving mRNA COVID-19 vaccines should receive 3 doses and a booster, consistent with CDC recommendations, practice nonpharmaceutical interventions, and, if infected, be monitored closely and considered early for proven therapies that can prevent severe outcomes.

## Footnotes

* Funded by CDC, the VISION Network includes Columbia University Irving Medical Center, New York, New York; HealthPartners Minnesota and Wisconsin; Intermountain Healthcare, Salt Lake City, Utah; Kaiser Permanente Northern California, Oakland, California; Kaiser Permanente Northwest, Portland, Oregon; Regenstrief Institute, Indianapolis, Indiana; and University of Colorado, Aurora, Colorado.

† Immunocompromised status was defined as the presence of at least one discharge diagnosis, using diagnosis codes from* International Classification of Diseases, Ninth Revision* (ICD-9) and *International Classification of Diseases, Tenth Revision* (ICD-10), for solid malignancy (ICD-10 codes: C00–C80, C7A, C7B, D3A, Z51.0, and Z51.1), hematologic malignancy (ICD-10 codes: C81–C86, C88, C90–C96, D46, D61.0, D70.0, D61.2, D61.9, and D71), rheumatologic or inflammatory disorder (ICD-10 codes: D86, E85 [except E85.0], G35, J67.9, L40.54, L40.59, L93.0, L93.2, L94, M05–M08, M30, M31.3, M31.5, M32–M34, M35.3, M35.8, M35.9, M46, and T78.40), other intrinsic immune condition or immunodeficiency (ICD-10 codes: D27.9, D61.09, D72.89, D80, D81 [except D81.3], D82–D84, D89 [except D89.2], K70.3, K70.4, K72, K74.3–K74.6 [except K74.60 and K74.69], N04, and R18), or organ or stem cell transplant (ICD-10 codes: T86 [except T86.82–T86.84, T86.89, and T86.9], D47.Z1, Z48.2, Z94, and Z98.85).

§ Index date was defined as the date of respiratory specimen collection associated with the most recent positive or negative SARS-CoV-2 test result before hospitalization or the hospitalization date if testing only occurred after admission.

¶ Hospitalizations with a discharge code consistent with COVID-19–like illness were included. COVID-19–like illness diagnoses included acute respiratory illness (e.g., COVID-19, respiratory failure, or pneumonia) or related signs or symptoms (cough, fever, dyspnea, vomiting, or diarrhea) using diagnosis codes from ICD-9 and ICD-10*.*

** 45 C.F.R. part 46; 21 C.F.R. part 56.
